# Old Age and Fermenlactyl

**Published:** 1908-05

**Authors:** 

**Affiliations:** Paris


### Old Age and Fermenlactyl.

By DR. SUZOR, Paris.

(*Written for the National Therapeutics.*)

OLD age and senility come to be a dreaded reality to every man and woman who has reached maturity; for the next step is death. It is easy, therefore, to understand the immense interest which has been universally attached to the recent researches of Professor Metchkinoff on the subject.

Exploding all the ancient notions and theories on senility, he was the first to look upon it as a chronic disease, in every way comparable to that developing in the course of various infections and intoxications. The cause of the infection here he found to be in the reabsorption of the ptomaines, conjugate sulphoether (indican, etc.) and other poisons formed by the bacteria of putrefaction which swarm in the large intestine. These poisons exert an irritating and exciting action which leads to the proliferation of large connective tissue cells, socalled giant cells or macrophagi. These, in normal, healthy life constitute the "phagocytes" of the same author and preserve our tissues from the invasion of germs of all sorts, which they arrest and destroy. In old age these same cells proliferate to excess, and attack the parenchymatous or specific cells of all our organs, brain, liver, kidney, bones, and the like, leading finally to the atrophy and degeneration of those organs.

Such being the theory, the practical conclusion is evident. It is possible to combat old age, to retard it, and to allow the human creature to reach death as a leaf does, without a pang, with the long familiar train of misery so common to old people. It is interesting, in this connection, to note that all animals which have, practically, no large intestine, birds, crocodiles, tortoises, etc., live to be very old, and preserve the appearance and attributes of youth to the very last.

"Fortune favors fools," still more does it favor the wise who have eyes to see. While traveling in Bulgaria and neighboring countries, Prof. Duclaux, late Director of the Pasteur Institute, and Prof. Metchnikoff were struck with the large proportion of strong, active old men whom they met. In fact, reliable statistics give, for a total population of about two millions and a half in Bulgaria, close upon three thousand centenarians, a good many of whom are over a hundred and twenty years old. The nature of the country itself and the general conditions of life of the natives does not appear to be able to account solely for this remarkable fact. One common custom, however, stood out conspicuously on examination. All the inhabitants live very largely on curdled sour milk called "yoghourt," prepared with a ferment called "maya." This ferment was found to be composed of a certain number of lactic acid-forming bacilli, the principal one of which is known as the "bacillus of Messol," from the Swiss doctor who first described it. It is of large size, measuring about 15 to 20 cm., and is characterised by the property it possesses of forming an unusually large proportion of lactic acid; when put in contact with saccharine substances, lactose, etc., as much as two per cent. When acting alone. however, it also decomposes fatty substances. thus communicating an unpleasant taste to milk. Hence its association, in practice, with various other lactic acid-forming bacilli, which have the property of restraining this fat-modifying action.

When absorbed with milk, or in the shape of a pure culture, this "bacillus of Messol" passes through the whole alimentary tract, and is found strong and active in the excreta. The lactic acid bacilli of Western countries are less resisting, and are no longer found in the same conditions; they are destroyed in the intestine. We may here remark that the ptomaines and other poisons referred to previously as being the primary cause of premature and morbid senility are all produced by the bacteria of putrid fermentation which swarm in the alkaline intestinal contents, and act there on the nitrogenous elements of food.

It is easy to understand, therefore, that the introduction of an acid in the intestines will tend to restrain this putrefactive action. The lactic acid bacilli, when carried into the intestine along with our food, continue to decompose sugary and starchy foodstuffs into lactic and succinic acids which, in their nascent state, are endowed with additional activity against the bacteria of putrefaction. Lesage and Hayem long ago showed the useful action of lactic acid in cases of infantile diarrhæa, enteritis, and even in cholera.

Experience has shown these lactic acid bacilli to exert (*a*) a local action on intestinal lesions (tuberculosis, etc.) ; (*b*) an antiseptic action on all putrefactive processes; (*c*) a reflex action on the liver and pancreas, the normal secretions of which are increased; (*d*) a general tonic action on the whole organism due to the lactic acid acting as such after resorption into the blood. Remarkable success has been obtained in the treatment of the following cases:

(1) Diseases of the digestive tract, such as gastroenteritis, the green summer diarrhea of infants, enterocolitis, diarrhea of hot countries, dysentery, intestinal tuberculosis, enteric fever.

(2) Whenever it becomes necessary to restrain to a minimum all causes of toxic action, *e. g.*, in cirrhosis of the liver, in all forms of chronic nephritis, Bright's disease, etc., in heart disease, arteriosclerosis.

(3) In many forms of skin diseases more or less directly dependent on the condition of the digestive system, eczema, urticaria or nettle rash, furuncles, and the like.

(4) In various states due to mechanical disturbance, hernia, intestinal ptosis, and the like.

(5) In various diseases due to a morbid modification of the hepatic or pancreatic secretions, *e.g.*, stone and gravel, pancreatic diabetes.

(6) In certain general diatheses, gout, rheumatism, arthritic diabetes.

(7) Whenever the patient, for some reason or other is submitted to a diet capable of promoting intestinal fermentation, *e.g.*, the nitrogenous overfeeding of tubercular patients; in infants fed on cow's milk, whether sterilised or not.

One of the best forms of presentation of the associated lactic acid bacilli, both as regards activity and good preservation, is the tablet form of the bacillus of Messol, etc. (fermenlactyl), prepared from cultures selected with great care at the Pasteur Vaccine Co's. Laboratories (Paris), with which the patient can prepare curdled milk at home. These tablets are best preserved in tubes hermetically sealed and kept dry and in a dark place. Fermenlactyl tablets advantageously replace kephyr, yoghourt, and all other lactic ferments.

